# Fever on hospital admission is associated with increased disease severity and mortality in acute pancreatitis: a systematic review and meta-analysis

**DOI:** 10.1186/s13643-026-03265-z

**Published:** 2026-07-21

**Authors:** Eszter Pakai, Krisztian Klonfar, Anna Walter, Nelli Farkas, Anna Sara Bognar, Jimin Lee, Peter Hegyi, Andras Garami

**Affiliations:** 1 Department of Thermophysiology, Institute for Translational Medicine, Medical School, University of Pecs, Pecs, Hungary; 2 Institute for Translational Medicine, Medical School, University of Pecs, Pecs, Hungary; 3 Institute of Bioanalysis, Medical School, University of Pecs, Pecs, Hungary; 4 Centre for Translational Medicine, Semmelweis University, Budapest, Hungary; 5 Division of Pancreatic Diseases, Heart and Vascular Center, Semmelweis University, Budapest, Hungary

**Keywords:** Acute pancreatitis, Body temperature, Fever, Meta-analysis, Mortality, Systemic inflammation

## Abstract

**Background:**

Acute pancreatitis (AP) is often accompanied by changes in body temperature (T_b_), but whether and how T_b_ at hospital admission is associated with the outcome of the disease remained unclear. Previous studies showed contradictory results regarding its prognostic value, and no meta-analysis was performed.

**Methods:**

In this systematic review and meta-analysis, we searched the PubMed, Embase, and CENTRAL databases (from inception to April 25, 2024) for studies in adult AP patients with reported admission T_b_ and mortality rate or a disease severity marker. With a random-effects model, we calculated pooled proportion (for mortality) or pooled mean (for secondary outcomes) with 95% CI and compared them between febrile and afebrile subgroups. With meta-regression, we assessed the correlation between admission T_b_ and mortality or length of hospital stay (LOS).

**Results:**

We found 26 eligible studies including data from 40,630 AP patients. The pooled mortality rate was higher (*p* < 0.001) in febrile patients (0.15, 95% CI 0.11–0.20; *I*^*2*^ = 40%) than in afebrile patients (0.05, 95% CI 0.03–0.09; *I*^*2*^ = 98%). LOS was longer (*p* = 0.025) in the febrile subgroup (39.1 days, 95% CI 16.8–61.4; *I*^*2*^ = 91%) than in the afebrile subgroup (17.5 days, 95% CI 7.6–27.5; *I*^*2*^ = 96%). With meta-regression, admission T_b_ significantly and positively correlated with mortality (0.09, 95% CI 0.07–0.12) and LOS (16.5, 95% CI 5.6–27.5). The APACHE II score was significantly (*p* = 0.030) higher, while the SOFA score tended to be higher (*p* = 0.168) in the febrile than in the afebrile subgroup. Blood levels of amylase and C-reactive protein did not differ meaningfully between the groups.

**Conclusions:**

Fever on admission is associated with higher mortality, increased disease severity, and longer hospitalization in AP. High-quality studies with standardized T_b_ measurements are needed to determine the prognostic performance of admission T_b_ in AP.

**Systematic review registration:**

This study was registered with PROSPERO on 16 January 2020 (registration number: CRD42020139154).

**Supplementary Information:**

The online version contains supplementary material available at https://doi.org/10.1186/s13643-026-03265-z.

## Background

Acute pancreatitis (AP) is one of the most common gastrointestinal disorders being responsible for hospitalization worldwide with a global incidence of 33.7 and 1.6 deaths per 100,000 person-years [1]. In the past decades, the incidence of AP as well as the disease-related complications have increased in developed countries [2–5], thereby continuing to pose a substantial burden on the patients, healthcare system, and economy [6, 7]. AP can present with varying degrees of severity, ranging from a mild disease with excellent recovery rates to severe forms. The overall mortality rate of mild AP is low (~1%), whereas in patients with severe forms of AP it may reach up to 35% (as reviewed by van Dijk and colleagues [8]), but even 40–45% according to some reports [9–11]. Clinical scoring systems are frequently used for prediction of severity in AP, but their clinical relevance is questionable because of their low predictive value [12]. Guidelines recommend monitoring indicators of systemic inflammation and organ failure [13, 14], but many questions remained regarding the most optimal treatment in AP [15]. A key mechanism in the progression of AP is the early development of systemic inflammation, involving the AP-associated inflammatory response and the compensatory activation of the anti-inflammatory response [16, 17], which can determine the severity as well as the outcome of the disease.

Systemic inflammation is a generalized pathological process, which can be clinically manifested in various forms, including sickness syndrome, systemic inflammatory response syndrome, sepsis, severe sepsis, septic shock, and multiple organ dysfunction syndrome [18, 19]. The inflammatory response is a series of complex pathophysiological events that can result from infections induced by microbial pathogens (e.g., sepsis, septic shock), as well as from non-infectious causes due to AP, stress, autoimmune diseases, trauma, surgery, burns, etc. In the non-infectious forms, systemic inflammation is induced by damage-associated molecular patterns (DAMPs) instead of pathogen-associated molecular patterns (PAMPs) that trigger the infectious forms [20]. After the insult, complex immune system alterations develop, which involve systemic proinflammatory reactions and compensatory anti‐inflammatory responses [21]. In AP, the pro- and anti-inflammatory responses arise in parallel [17], and maintain a delicate balance during different phases of the disease [22].

The development of systemic inflammation is often accompanied by changes in the regulation of deep body temperature (T_b_), which can be manifested as fever or hypothermia [19]. In infection-related systemic inflammation (i.e., sepsis), the direction of the change in T_b_ is associated with the outcome of the disease, since fever at hospital admission predicts decreased, whereas hypothermia increased death in sepsis [23–25]. However, in AP, which is a form of non-infectious systemic inflammation, the significance of deep T_b_ alterations at hospital admission has remained controversial in human patients. In some studies, fever was associated with high mortality (≥ 20%) [26–28], whereas in other reports the mortality was only 5–6% in febrile AP patient groups [29–31]. Furthermore, in normothermic patients with AP, groups with different severities, thus different mortality rates (0–17%) were reported [32, 33]. We could not find any previous meta-analysis which attempted to quantitatively summarize the available literature data. Hence, it has remained unclear whether deep T_b_ on hospital admission is associated with the severity and the outcome of AP, therefore, if deep T_b_ can be used as a prognostic biomarker of the disease.

In the present meta-analysis, for the first time to our knowledge, our aim was to identify the significance of deep T_b_ on hospital admission as a predicting factor of the severity and outcome of AP by analyzing the available literature data from human studies.

## Methods

### Search strategy and selection criteria

Our meta-analysis was conducted following the guidelines of the Preferred Reporting Items for Systematic Reviews and Meta-Analyses (PRISMA) protocols [34] (Table S1), similarly, as in our previous studies [35, 36].

Our clinical question was raised in the PICO (Population, Intervention, Control, Outcome) format: in adult patients with AP we aimed to determine the predictive role of the alterations of deep T_b_ at hospital admission on mortality as the primary outcome, as well as on secondary outcomes such as length of hospital stay (LOS), clinical severity scores [Acute Physiology and Chronic Health Evaluation (APACHE II) and Sequential Organ Failure Assessment (SOFA)], and blood parameters [serum amylase and C-reactive protein (CRP) levels].

A systemic search of medical databases, including PubMed (MEDLINE), Embase, and the Cochrane Central Register of Controlled Trials, was performed for human observations, from inception to April 25, 2024, using the following search key: “pancreatitis AND (“body temperature” OR fever OR hypothermia OR hyperthermia)”.

EndNote 20.4 bibliography management software (Clarivate Analytics, Philadelphia, PA, USA) was used to catalog all the identified articles. After removing the duplicates, two independent investigators (EP and KK) screened the titles and abstracts for study eligibility. Any disagreements were resolved by consensus with the help of a third party (AG).

We included all studies in adult patients with AP, wherein deep T_b_ was measured and reported on hospital admission, and at least one of the abovementioned outcomes of our interest was published. During the selection process, reviews, case reports, and abstracts with incomplete data reporting were excluded. Studies conducted in pediatric AP patients and in adult patients with chronic pancreatitis at the acute-on stage or recurrent AP were excluded, as well as reports describing novel coronavirus infection (COVID-19)-related AP. Articles that fulfilled the inclusion criteria were assessed for full-text evaluation.

### Data analysis

Data of interest from eligible studies were extracted into Microsoft Excel (version 16.0) by EP and double-checked by KK. From all included articles, we extracted the study design, the sample size, sex ratio, and age, the country of origin, the reported mean T_b_ for the studied patient groups with the corresponding standard deviation (SD), as well as the mortality ratio, clinical severity scores, LOS, and serum amylase and CRP levels within the groups. From two articles, which presented the data in figures with linear scales [37, 38], the T_b_ and serum amylase values were extracted with WebPlotDigitizer application. Since these papers presented the serum amylase data in Somogyi units, they were converted to U/L [37, 38], while T_b_ values were converted from Fahrenheit to Celsius degrees in one of them [37].

When the SD was not reported in a study, but the standard error (SE) was available, the SD was calculated by multiplying SE with the square root of the sample size. When the median and the range or its indicator (e.g., quartiles or percentiles) were reported, then the mean and the SD were estimated with the methods described by Lou [39] and Shi [40], as implemented in the used meta R package.

To analyze the influence of the admission T_b_ on the severity and the outcome of AP, from each study we assigned the patient groups based on the reported T_b_ on hospital admission into nonfebrile (below 37.5°C) or febrile (37.5°C and above) populations, irrespective from the original basis for grouping used by the authors of the study. In two articles [30, 41], we used subgroups labeled as “a” and “b,” because the patient subgroups in those studies belonged to different T_b_ groups (viz*.*, febrile vs. nonfebrile). The 37.5°C T_b_ threshold for the separation of the groups was selected prior to our analyses based on expert opinions in the field [42–44].

All statistical analyses were performed with R (R Core Team 2023, v4.3.0) using the “meta” package for basic meta-analysis calculations and plots (v6.2.1, 2023) [45], and the “dmetar” package (v0.0.9000) for additional influential analysis calculations and plots. Random-effects model based on the Hartung-Knapp-Sidik-Jonkman method [46] was used to pool effect sizes. Depending on the analyzed outcome, ratio (for mortality) or mean (for LOS, clinical scores, and serum levels) with 95% confidence interval (CI) were used for the effect size measure and presented as “forest plots.” Meta-regression analyses were performed to further explore the associations between T_b_ and the outcomes of interest. A *p* value of less than 0.05 was considered as significant difference.

Small study publication bias was assessed by visual inspection of funnel plots and by calculating the Egger’s test (Egger’s test values of less than 0.1 were considered as indicators of significant small-study effect) or Peters (modified Egger’s) test *p* value [47]. Between-study heterogeneity was assessed with the *I*^*2*^ statistical test [48], where *I*^*2*^ is the proportion of total variation attributable to between-study variability (an *I*^*2*^ value of more than 50% was considered as an indication of substantial heterogeneity), as in our previous meta-analyses [35, 36]. Sensitivity analysis (i.e., iteratively omitting one study from the analyses and recalculating the pooled effect size measure to investigate the impact of the individual study on the summary estimate) was performed to test the impact of the individual studies.

To evaluate the quality of the included studies, two authors (ASB and JL) independently assessed the risk of bias of each study with the Joanna Briggs Institute (JBI) Critical Appraisal Checklist for Cohort Studies in the case of comparative studies [49], and the JBI Critical Appraisal Checklist for Case Series in the cases of single-arm studies [50]. Any disagreements were resolved by consensus.

This meta-analysis was registered with PROSPERO (registration number: CRD42020139154).

## Results

**Fig. 1:**
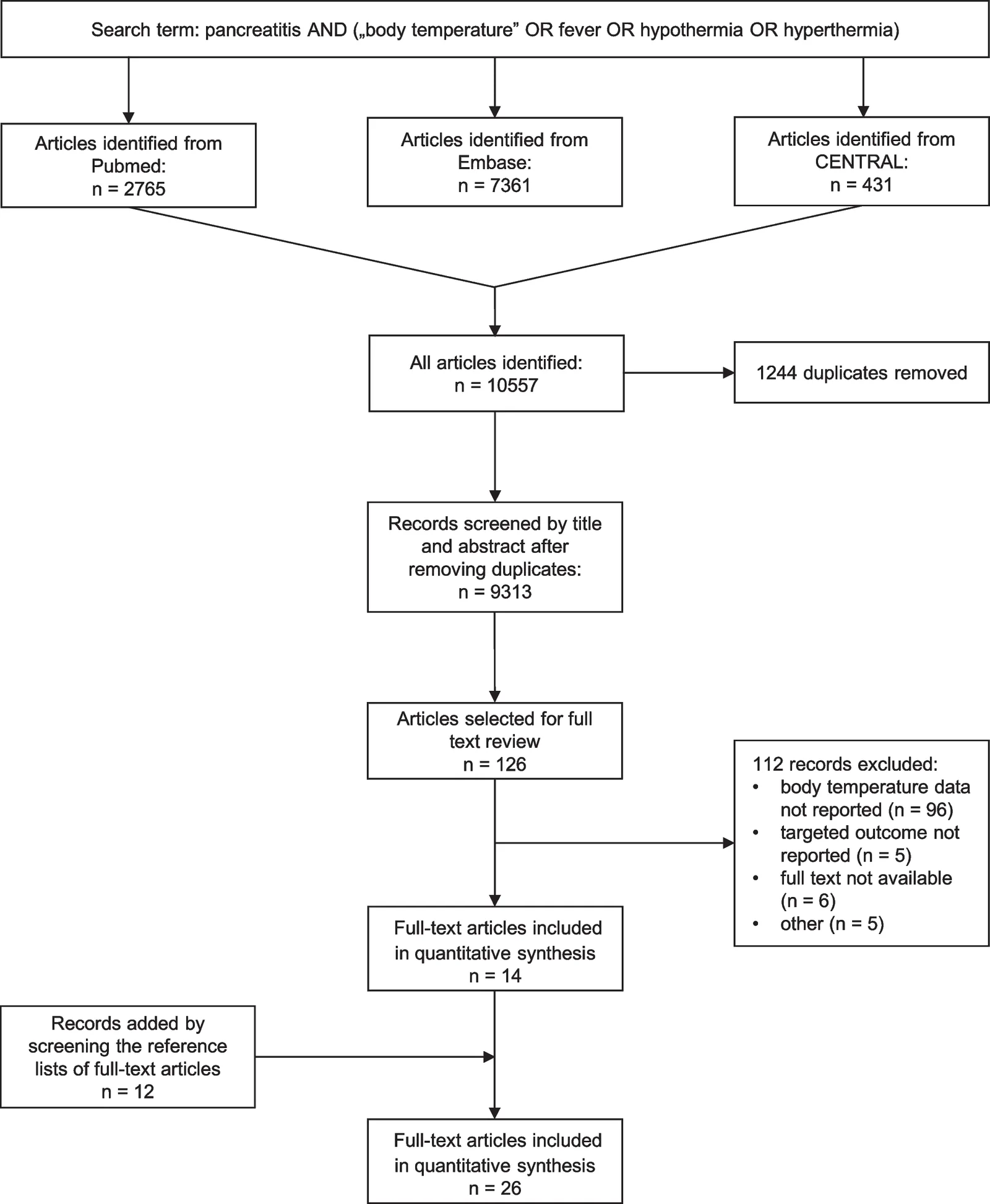
Study selection

The literature search of the 3 databases identified altogether 10,557 articles, of which 1244 were duplicates, as presented on the flow chart of the study selection (Fig. 1). After removing duplicates, 9313 records were screened by title and abstract. From those, 126 papers were eligible for evaluation of the full text; however, 112 articles had to be excluded, because the T_b_ values were not reported (*n* = 96), the targeted outcomes were not revealed (*n* = 5), the full text of the paper was not obtainable (*n* = 6), and other reasons (*n* = 5), such as pediatric patients, recurrent AP patients, or not suitable data after translation to English. Thereby, 14 full-text publications fulfilled the eligibility criteria for statistical analysis. By the manual search of the reference lists of the eligible articles, we identified and retrieved 12 additional studies, which increased the total number of eligible studies to 26 [26–33, 37, 38, 41, 51–65]. These studies included data from a total of 40,630 adult patients with AP.

The descriptive characteristics of the patient groups from the selected studies are shown in Table 1, which includes the country of origin, study design and period, characteristics of the population (sample size, age, sex ratio), deep T_b_ on hospital admission, AP severity ratios, and the mortality rate from each study. The majority of the studies (23 out of 26) were published after 1992, thus after the first Atlanta classification of AP [66], from which 15 studies are dated later than the revision of the Atlanta classification in 2012 [67]. Among the included trials, 22 were conducted in a single center [26–33, 37, 38, 51–55, 57–59, 61–63, 65], while 4 were multicenter studies [41, 56, 60, 64]. The ranges for the number of patients, age, and proportion of females were 13–36,178, 18–108 years, and 19–62%, respectively.

**Table 1:** Summary of study characteristics included in the meta-analysis

First author (year)	Country	Study design	Study period	AP severity (%)	Patients N (males)	Age (years)^a^	Clinical severity score^b^	T_b_ (°C)^a^	Deaths *n* (%)
Andresson et al. (2011) [51]	Sweden	PS	2002–2005	Mild (85.6) Severe (14.4)	139 (75)	61 (20)	7.3 (6.8–7.8)	37.3 (0.9)	NR
Biberoglu et al. (2017) [32]	Turkey	RS	2010–2011	Mild (42.1) Moderate (42.1) Severe (15.8)	76 (29)	Range (18–60+)	NR	36.8 (0.5)	2 (2.6)
Chu et al. (2013) [26]	China	PRS	2005–2010	Severe (100.0)	30 (15)	43 (15)	22.7 (21.0–24.4)	38.7 (0.7)	8 (26.7)
Davis et al. (2013) [27]	Canada	RS	2005–2009	Severe (100.0)	45 (36)	59 (13)	20.3 (18.4–22.2)	37.8 (0.7)	11 (24.4)
Dickerson et al. (1991) [52]	USA	PS	NR	NR	13 (7)	52 (22)	NR	37.2 (0.7)	NR
Fan et al. (1993) [53]	Hong Kong	PRS	1988–1991	Mild (58.5) Severe (41.5)	195 (80)	Range (17–94)	Ranson’s score: median (range) 3 (0–8) 3 (0–9)	37.0 (0.8)	14 (7.2)
Hedjoudje et al. (2021) [54]	France	RS	2001–2012^c^	NR	499 (277)	NR	SOFA: 4.5 (4.1–4.8)	37.1 (0.7)	46 (9.2)
Hökenek et al. (2023) [55]	Turkey	RS	2015–2020	NR	143 (61)	62 (16)	CTSI: Mean (SD) 2.3 (1.4)	36.3 (0.7)	13 (9.1)
Kneisel et al. (1968) [37]	USA	PS	NR	NR	23 (NR)	NR	NR	38.0 (0.9)	4 (17.4)
Kui et al. (2022) [56]	15 countries	PS	NR	Mild (74.2) Moderate (20.5) Severe (5.3)	1184 (688)	56 (17)	NR	36.7 (0.5)	26 (2.2)
Liu et al. (2022) [33]	China	RS	2001-2012^c^	NR	631 (348)	Median (25th; 75th percentile) 60 (47; 73)	SOFA: 5.4 (3.2–7.6)	36.9 (1.1)	88 (13.9)
Lu et al. (2022) [57]	China	RS	2014–2020	Severe (100.0)	98 (69)	46 (14)	CTSI: Median (range) 8 (4–10); 8 (2–10)	37.0 (0.9)	12 (12.2)
Martinez et al. (1984) [38]	Spain	PRS	1982	Severe (100.0)	31 (25)	48 (16)	NR	37.8 (0.8)	5 (16.1)
Quan et al. (2003) [58]	China	PS	1998–2002	Severe (100.0)	14 (8)	41 (16)	NR	38.2 (0.6)	0 (0.0)
Talamini et al. (1996) [59]	Italy	RS	1976–1992	NR	192 (117)	Median (25th; 75th percentile) 49 (35; 59) 60 (47; 67)	NR	36.8 (2.3)	17 (8.9)
Tu et al. (2013) [29]	China	RS	2006–2012	Severe (100.0)	50 (28)	Range (30–78)	13.4 (5.9–20.9)	38.8 (4.6)	5 (10.0)
van Grinsven et al. (2017) [60]	Netherlands	RS	2004–2014	Severe (100.0)	117 (78)	62 (15)	CTSI: Median (IQR) 10 (9–10) 10 (8–10)	38.2 (1.3)	21 (18.0)
van Santvoort et al. (2009a) [41]	Netherlands	PS	2004–2007	NR	75 (34)	65 (16)	8.7 (7.9–9.5)	37.8 (0.9)	12 (16.0)
van Santvoort et al. (2009b) [41]	Netherlands	PS	2004–2007	NR	78 (38)	65 (15)	8.6 (7.8–9.5)	37.4 (0.7)	7 (9.0)
Vogel et al. (2022) [61]	Germany	RS	2011–2020	NR	104 (74)	Median (IQR): 49 (43–60)	SOFA: 0.4 (0.2–0.5)	36.3 (0.5)	2 (1.9)
Wang et al. (2003) [62]	China	PS	1997–2002	Severe (100.0)	28 (21)	51 (13)	14.4 (12.9–15.8)	37.8 (0.8)	6 (21.4)
Wang et al. (2018) [63]	China	PS	2011–2015	Severe (100.0)	260 (172)	45 (13)	8.8 (8.2–9.3)	36.6 (1.6)	NR
Wang et al. (2021a) [30]	China	PS	2019–2020	Moderate (57.5) Severe (42.5)	73 (44)	Median (25th; 75th percentile) 48 (34; 60) 44 (35; 50)	8.4 (6.0–10.9)	38.4 (1.2)	5 (6.9)
Wang et al. (2021b) [30]	China	PS	2019–2020	Mild (100.0)	30 (16)	Median (25th; 75th percentile) 53 (36; 63)	4.0 (2.2–5.8)	37.3 (0.8)	0 (0.0)
Wu et al. (2008) [64]	USA	RS	2000–2001	NR	36,178 (18,069)	Range (18–108)	NR	36.3 (0.8)	574 (1.6)
Zhang et al. (2020) [65]	China	RS	2014–2018	NR	92 (59)	46 (15)	16.3 (14.9–17.7)	37.1 (0.4)	NR
Zhifeng et al. (2020) [31]	China	RS	2007–2017	NR	169 (115)	50 (14)	15.1 (13.9–16.3)	37.6 (0.8)	15 (8.9)
Zhu et al. (2009) [28]	China	RS	2005–2007	Severe (100.0)	63 (37)	55 (13)	15.6 (14.6–16.6)	38.2 (0.8)	13 (20.6)

^a^Data are reported as mean (SD) unless specified otherwise

^b^Mean APACHE II scores (95% CI) are reported unless specified otherwise

^c^Data were assessed from Medical Information Mart for Intensive Care III (MIMIC-III) database

*AP *acute pancreatitis, *APACHE *Acute Physiology and Chronic Health Evaluation, *CTSI *Computed Tomography Severity Index, *NR *not reported, *PS *prospective study, *PRS* prospective randomized study, *RS *retrospective study, *SOFA *Sequential Organ Failure Assessment, *T*_*b*_ body temperature

**Fig. 2:**
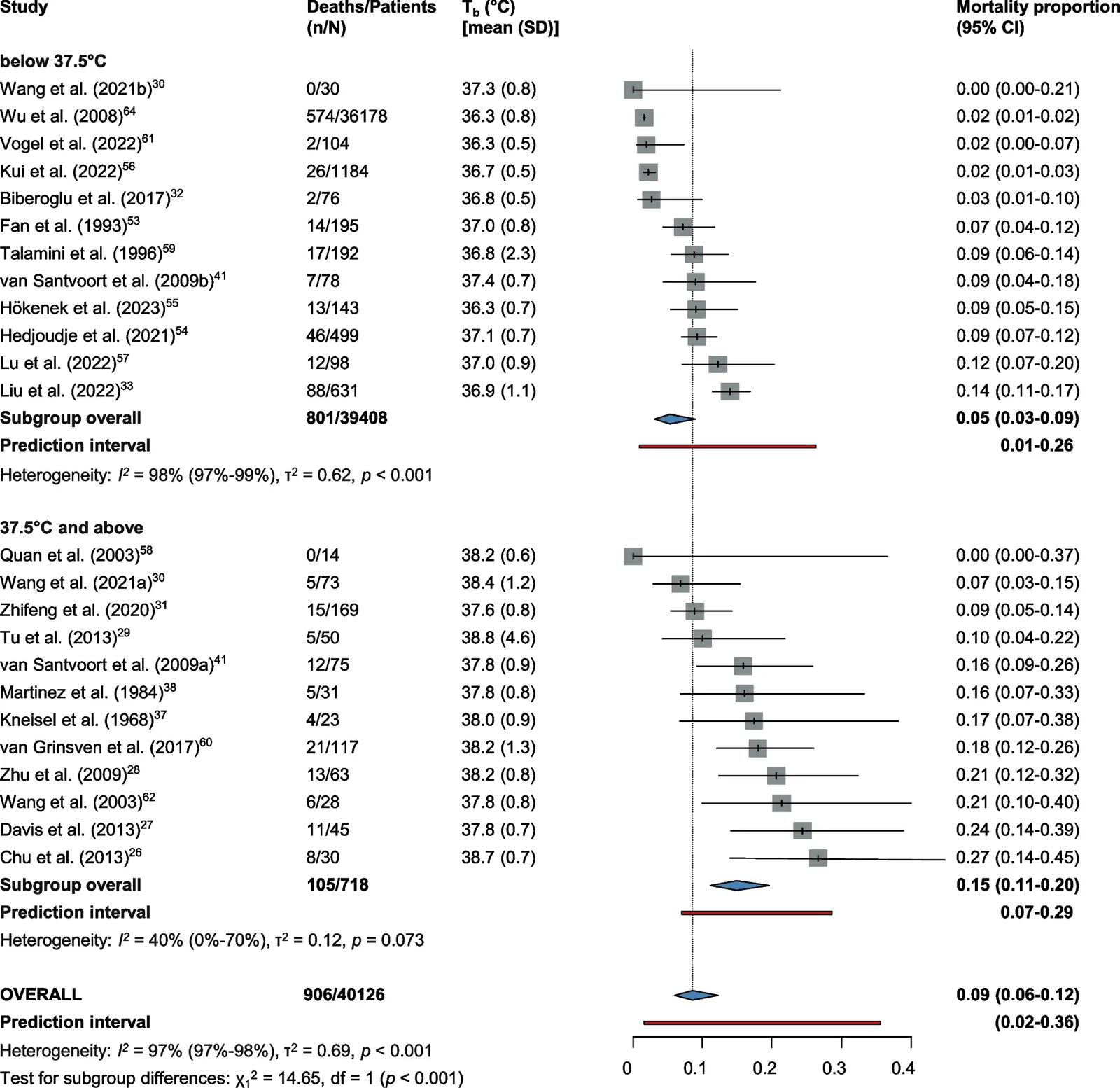
Forest plot of mortality rates in febrile and afebrile subgroups of AP patients. Here, and in Figs. 4, 6, 7, 8, and 9 the centers of the crosshairs represent the mortality rate or a mean outcome parameter for each study, while the left and right horizontal arms indicate the corresponding 95% CI. The size of the gray box surrounding the crosshair is proportional to the relative weight of the study. The blue diamonds represent the pooled mortality rate calculated from the mortality rates of the individual studies in a subgroup (top and middle) and in all studies (bottom). The left and right vertices of the diamonds represent the 95% CI of the pooled mortality rate. The vertical dashed line is determined by the low and top vertices of the bottom diamond and indicates the value of the pooled mortality rate of all studies in the forest plot. The red lines below the subgroups and on the bottom of the forest plot indicate the prediction intervals

After structuring the extracted data, we used a multiple approach to identify any potential association between T_b_ and disease severity. First, we compared the primary outcome, mortality rate, between AP patients with and without fever. In 22 studies, we found 12 febrile AP patient groups [26–31, 37, 38, 41, 58, 60, 62], in which mean T_b_ was reported as 37.5°C or above (*N* = 718), while another 12 groups were afebrile [30, 32, 33, 41, 53–57, 59, 61, 64] (mean T_b_ < 37.5°C) (*N* = 39,408) (Fig. 2). The pooled mortality rate of the febrile patient groups was 0.15 (95% CI 0.11–0.20; *I*^*2*^ = 40%), which was significantly (*p* < 0.001) higher than the pooled mortality rate of 0.05 (95% CI 0.03–0.09; *I*^*2*^ = 98%) in the afebrile groups. The overall pooled mortality rate, including both febrile and afebrile patient groups (*N* = 40,126) was 0.09 (95% CI 0.06–0.12), with substantial heterogeneity (*I*^*2*^ = 97%). Funnel plot asymmetry suggested publication bias, which was also indicated by the Peters’ test (*p* = 0.0009) (Fig. S1). Sensitivity analysis revealed that the overall pooled mortality rate did not vary substantially after excluding any individual study (Fig. S2), indicating that the results were not driven by one of the analyzed individual studies. With regards to heterogeneity, omitting the study by Wu et al. [64] decreased the overall heterogeneity from 97 to 83% (Fig. S2), which still remained substantial, while omitting any other studies did not have an influential effect.

**Fig. 3:**
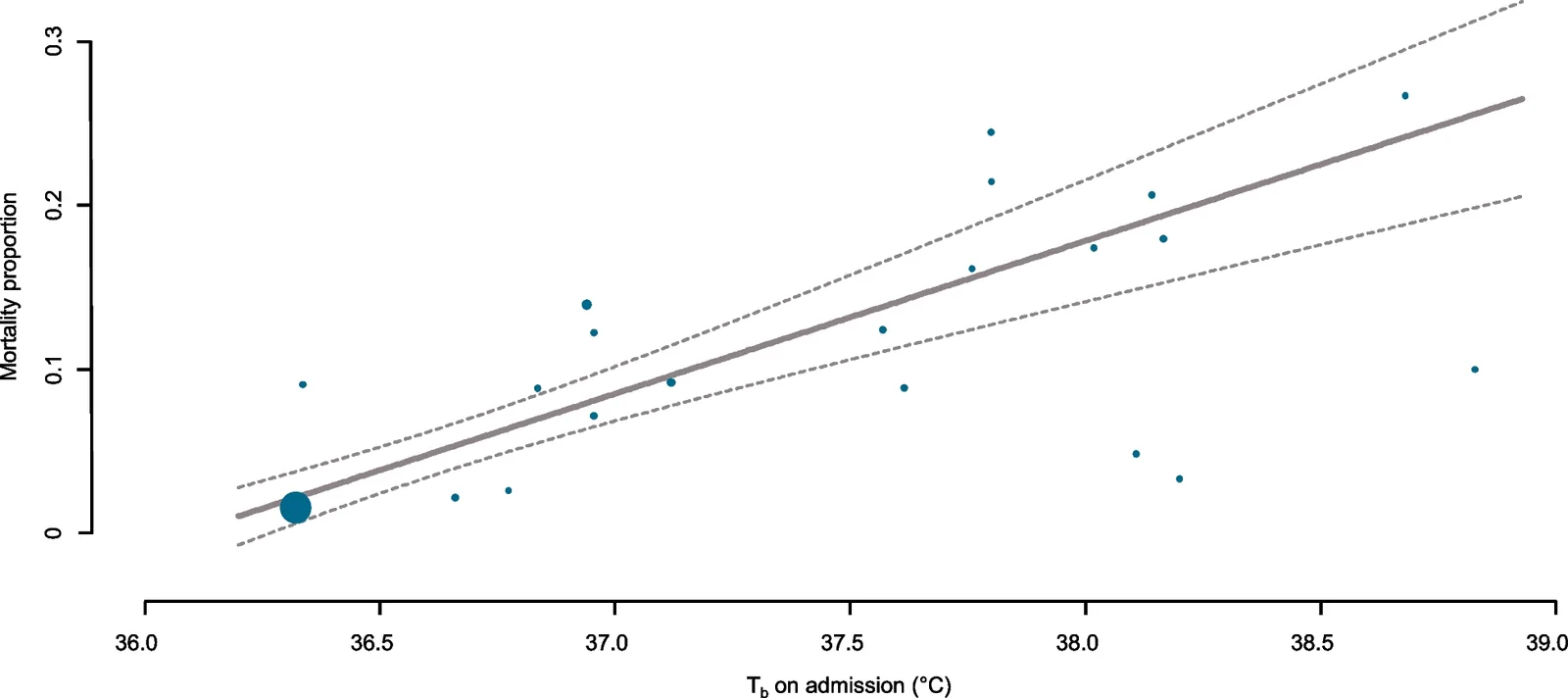
Meta-regression of the association between T_b_ on hospital admission and mortality rate of AP patients. Here and in Fig. 5, the circles indicate the mortality rate or LOS, respectively, of each patient group. The circle size is proportional to the precision of the estimated mortality rate or LOS. The solid black line represents the weighted regression line based on variance-weighted least squares, while the dashed lines indicate the 95% CI

While the selection of the high vs. normal T_b_ threshold was based on expert opinions in our analysis [68], it might be criticized. Therefore, instead of selecting other thresholds, in our next approach we performed a meta-regression analysis, which omits the need for thresholds in order to observe the association between T_b_ and mortality, as a primary outcome (Fig. 3). We used those study groups, in which T_b_ and mortality rate was reported in AP for the same patient groups. We found a significant (*p* < 0.001) positive linear correlation between T_b_ and mortality rate with an estimated T_b_ effect (regression slope) of 0.0924 (95% CI 0.0675–0.1173), indicating that mortality rate increases on average by 9% for each degree rise of T_b_. Importantly, sensitivity analysis revealed that the overall pooled mortality rate calculated from all 22 studies included in the meta-regression analysis did not vary substantially after excluding any individual study, indicating that the results were not driven by one of the analyzed individual studies. For example, exclusion of the study by Chu et al. [26], which is indicated by the top right data point in Fig. 3, resulted in an estimated T_b_ effect of 0.0917 (95% CI 0.0646–0.1189; *p* < 0.001).

**Fig. 4:**
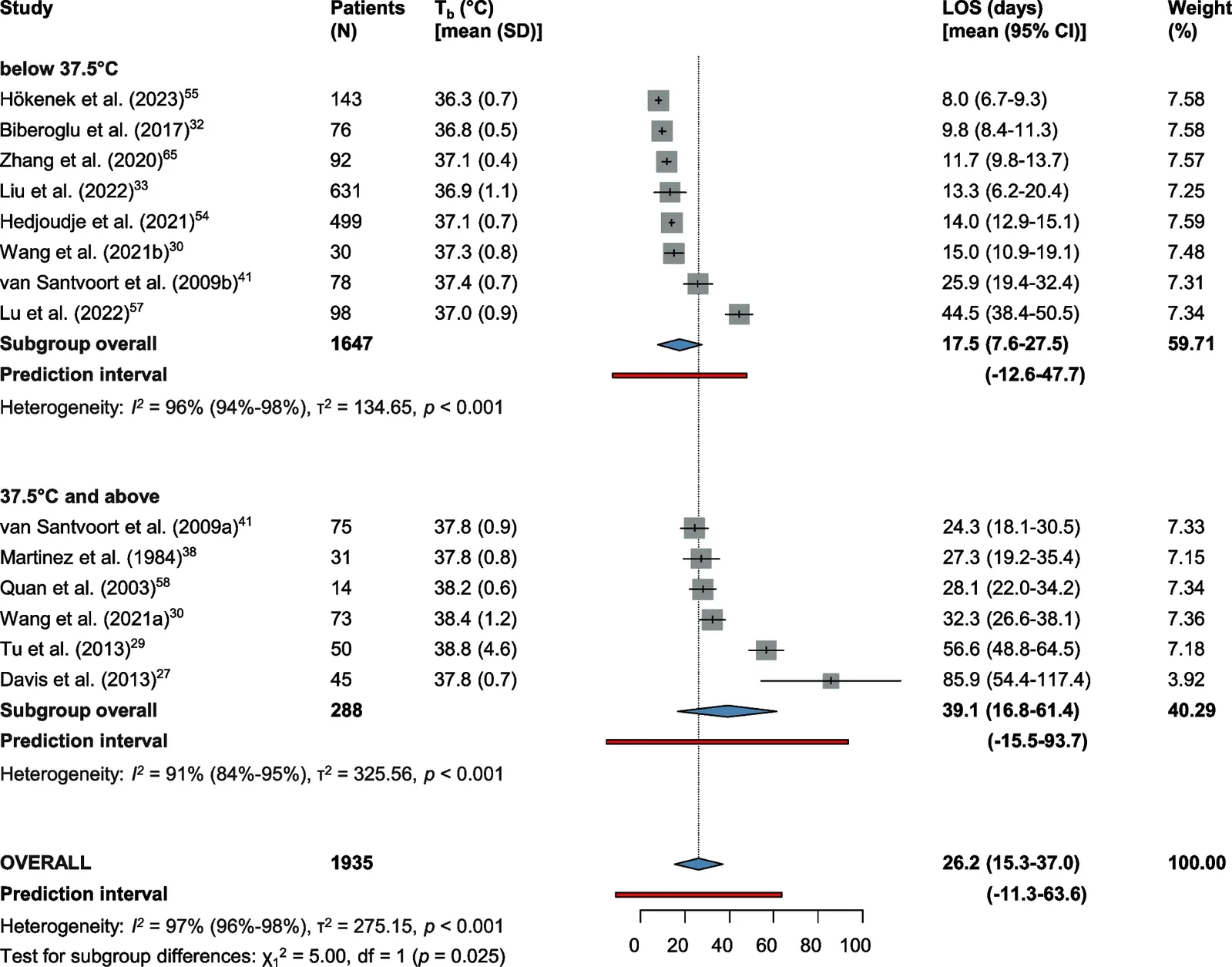
Forest plot of LOS in febrile and afebrile subgroups of AP patients

**Fig. 5:**
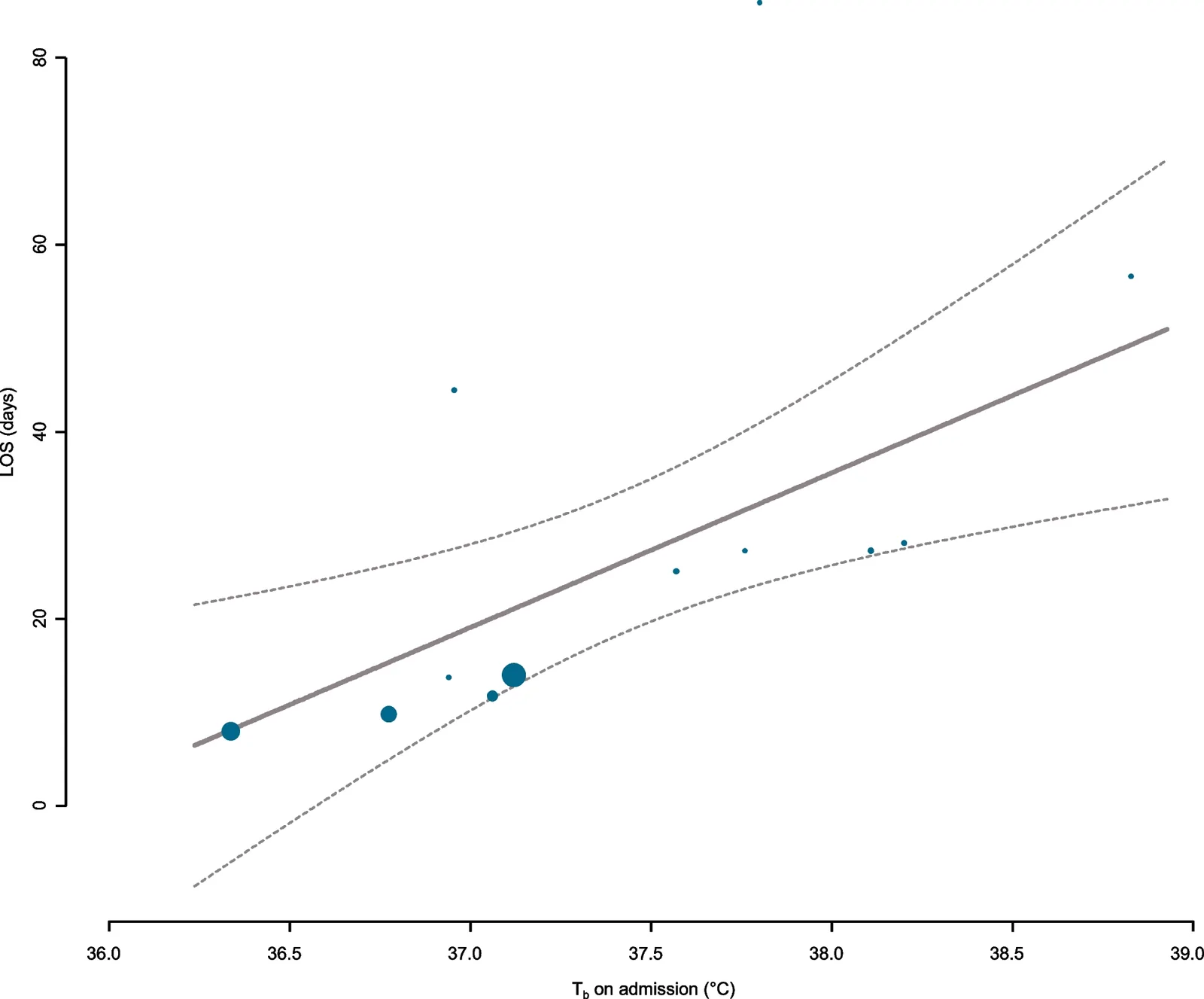
Meta-regression of the association between T_b_ on hospital admission and LOS of AP patients

Next, we analyzed the association of T_b_ with the secondary outcomes. Similarly, as in the case of mortality, first we compared the LOS between febrile and afebrile patient groups (Fig. 4). We found that AP patients in the febrile groups (*N* = 288 from 6 studies) required 39.1 days (95% CI 16.8–61.4; *I*^*2*^ = 91%) of hospitalization, which was more than two times longer than the 17.5 days (95% CI 7.6–27.5; *I*^*2*^ = 96%) of LOS in the afebrile patient groups (*N* = 1647 from 8 studies). The difference in LOS was statistically significant between the two T_b_ groups (*p* = 0.025). The overall pooled LOS of all (febrile and afebrile) patient groups (*N* = 1935) was 26.2 (95% CI 15.3–37.0) with substantial heterogeneity (*I*^*2*^ = 97%). Funnel plot asymmetry suggested publication bias, which was also indicated by the Egger’s test (*p* = 0.0014) (Fig. S3). Sensitivity analysis revealed that the overall pooled LOS and heterogeneity did not vary significantly after excluding any individual study (Fig. S4), indicating that the results were not driven by one of the analyzed individual studies. Meta-regression showed a significant (*p* = 0.0031) positive linear correlation between T_b_ and LOS with a regression slope of 16.5301 (95% CI 5.5727–27.4875) (Fig. 5). These findings suggest that for every degree increase of T_b_ on hospital admission on average more than 2 weeks longer hospitalization can be expected.

**Fig. 6:**
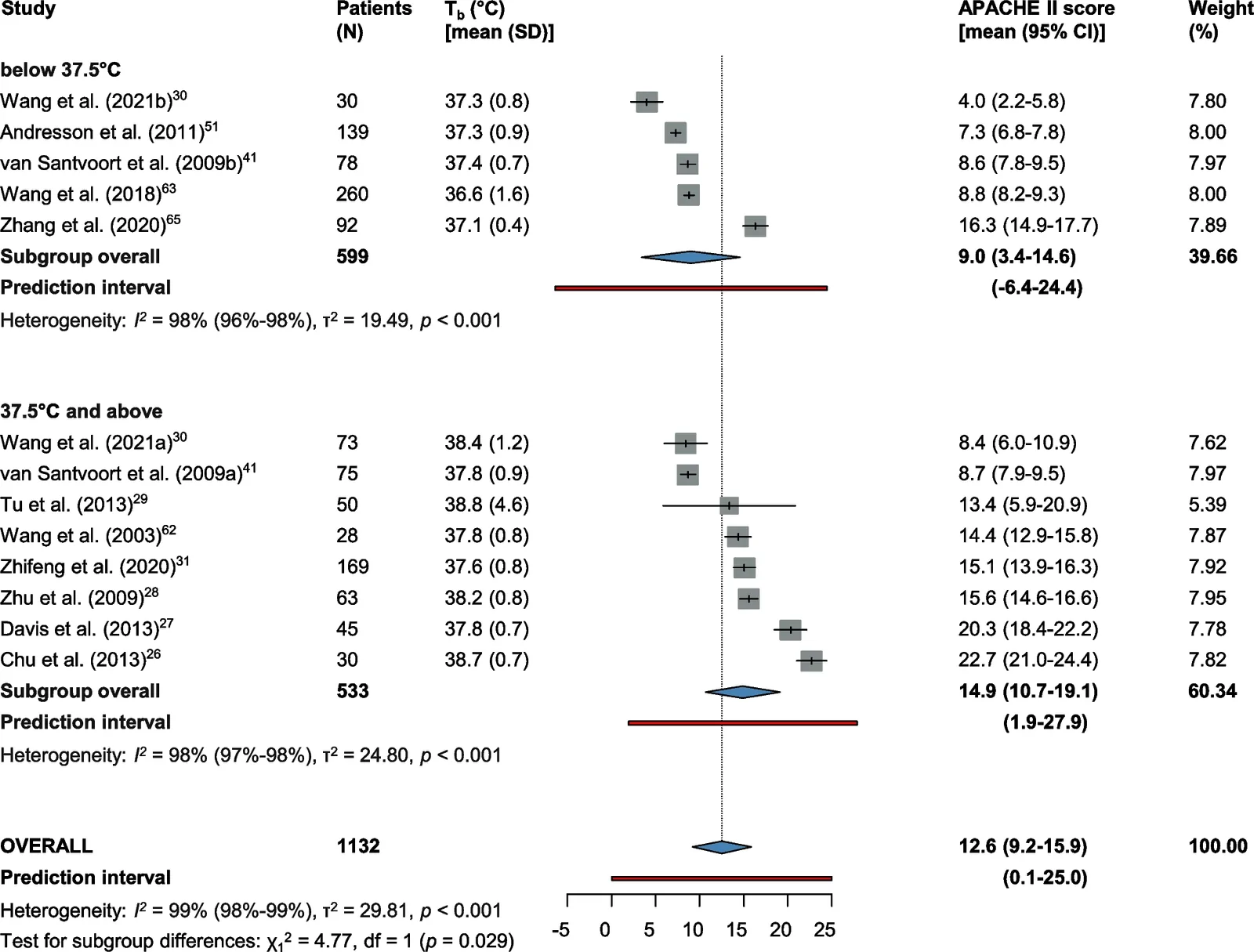
Forest plot of APACHE II scores in febrile and afebrile subgroups of AP patients

**Fig. 7:**
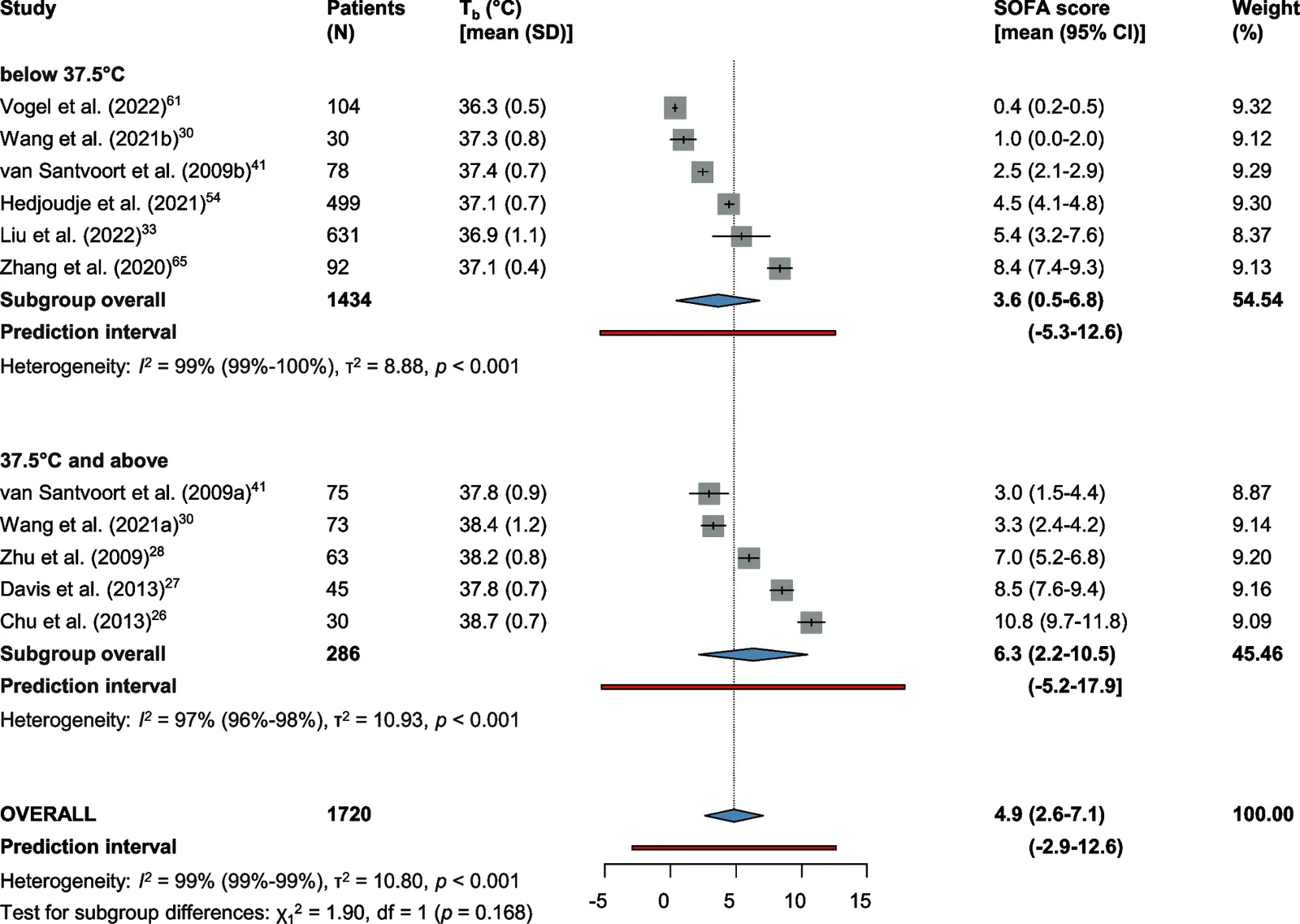
Forest plot of SOFA scores in febrile and afebrile subgroups of AP patients

We also wanted to know if higher T_b_ on admission is associated with elevated clinical severity scores. We found available data about 2 scores (APACHE II and SOFA) that could be included in our analyses (Figs. 6 and 7). In the febrile patient groups (*N* = 533), the pooled APACHE II score was 14.9 (95% CI 10.7–19.1; *I*^*2*^ = 98%), while in the afebrile patient groups (*N* = 599) it was 9.0 (95% CI 3.4–14.6;* I*^*2*^ = 98%) with significant difference between the two subgroups (*p* = 0.029) (Fig. 6). The overall pooled APACHE II score from all included AP patient groups (*N* = 1132) was 12.6 (95% CI 9.2–15.9), with substantial heterogeneity (*I*^*2*^ = 99%). With regards to SOFA, in febrile patient groups (*N* = 286) the pooled score was 6.3 (95% CI 2.2–10.5; *I*^*2*^ = 97%), while in the afebrile patient groups (*N* = 1434) it was 3.6 (95% CI 0.5–6.8; *I*^*2*^ = 99%), which tended to be lower, but the difference was not statistically different between the subgroups (*p* = 0.168) (Fig. 7). The pooled SOFA score from all patients (*N* = 1720) was 4.9 (95% CI 2.6–7.1), again with substantial heterogeneity (*I*^*2*^ = 99%). Funnel plot asymmetry suggested publication bias, which was also indicated by the Egger’s test for both APACHE II and SOFA score analysis (*p* = 0.0483 and *p* = 0.0077, respectively) (Figs. S5–6). Sensitivity analysis revealed that the overall pooled APACHE II and SOFA scores and heterogeneities did not vary meaningfully after excluding any individual study (Figs. S7–8).

**Fig. 8:**
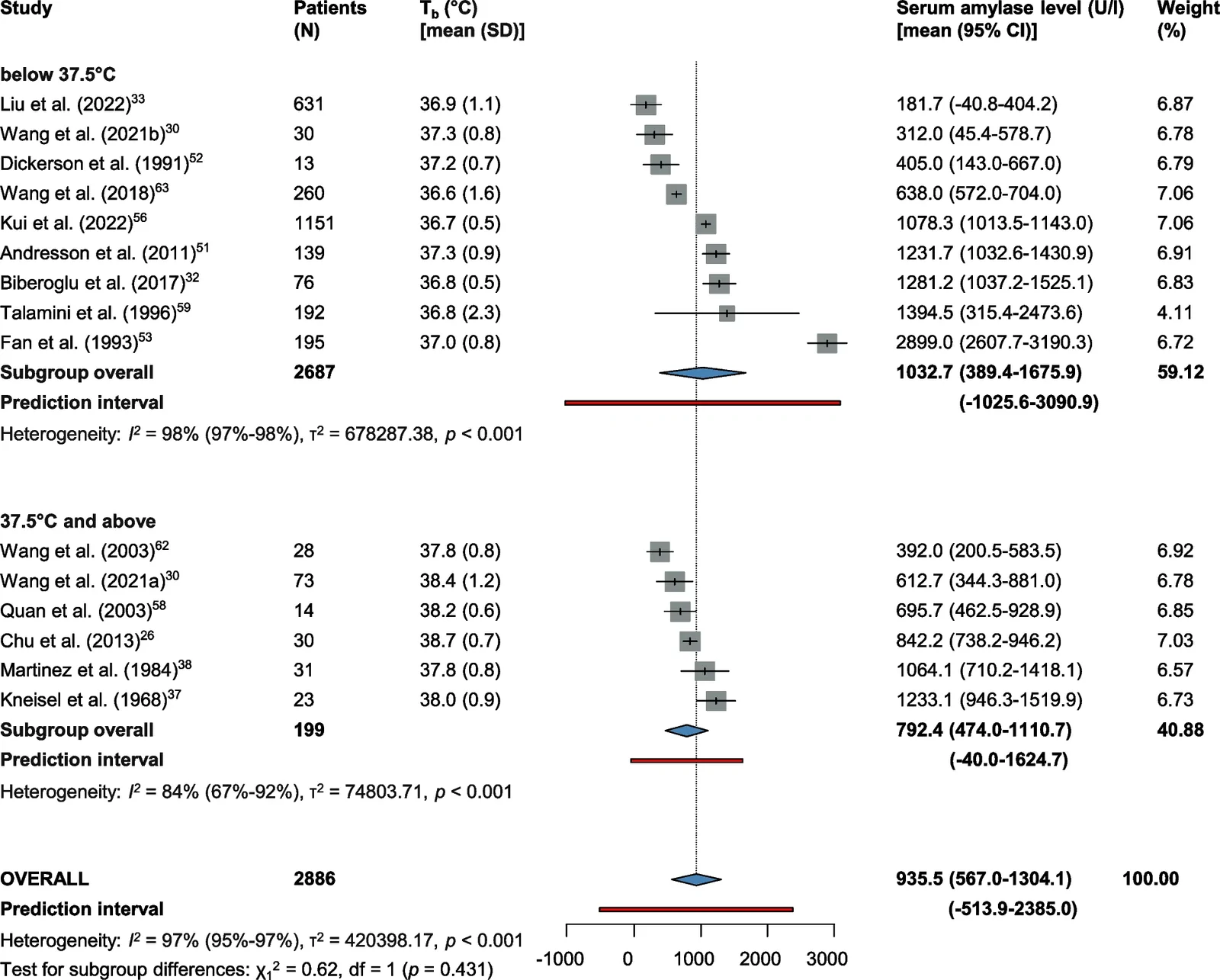
Forest plot of serum amylase levels in febrile and afebrile subgroups of AP patients

**Fig. 9:**
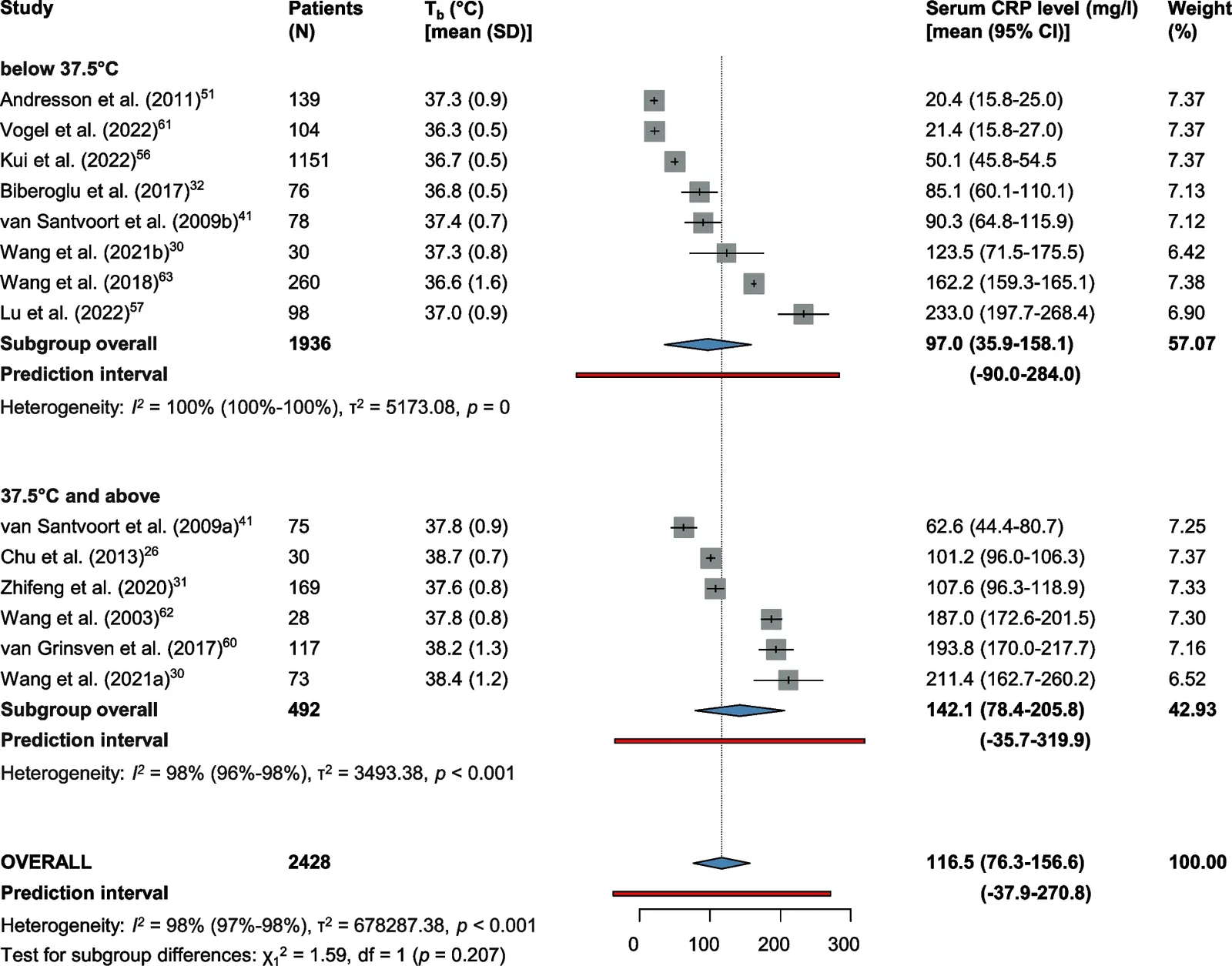
Forest plot of serum CRP levels in febrile and afebrile subgroups of AP patients

Last, we analyzed whether admission T_b_ is associated with classic blood biomarkers of AP and systemic inflammation: serum amylase and CRP levels, respectively (Figs. 8 and 9). We did not find a meaningful difference (*p* = 0.431) in serum amylase levels between the febrile (*N* = 199) and afebrile (*N* = 2687) groups: 792.4 U/l (95% CI 474.0–1110.7; *I*^*2*^ = 84%) vs. 1032.7 U/l (95% CI 389.4–1675.9; *I*^*2*^ = 98%) (Fig. 8). The overall pooled amylase level was 935.5 U/l (95% CI 567.0–1304.1) with substantial heterogeneity (*I*^*2*^ = 97%). We also did not find a difference (*p* = 0.207) in the serum CRP levels between the febrile (*N* = 492) and afebrile (*N* = 1936) subgroups: 142.1 mg/l (95% CI 78.4–205.8; *I*^*2*^ = 98%) vs. 97.0 mg/l (95% CI 35.9–158.1; *I*^*2*^ = 100%) (Fig. 9). The overall pooled CRP level was 116.5 mg/l (95% CI 76.3–156.6) with substantial heterogeneity (*I*^*2*^ = 98%). There was no meaningful funnel plot asymmetry to suggest publication bias or other small study effects in case of either amylase or CRP level analysis (Egger’s test *p* = 0.7644 and *p* = 0.9045, respectively) (Figs. S9–10). In case of serum amylase, the sensitivity analysis revealed that omitting the study by Fan et al. [53] decreased the overall pooled amylase level from 935.5 U/l to 781.2 U/l and the heterogeneity from 97 to 94% (Fig. S11), while omitting any other studies did not have a meaningful effect. It should be noted, however, that by omitting the study by Fan et al. [53] from the analyses, the pooled subgroup serum amylase levels would become even closer to each other and heterogeneity would still remain substantial, thus no meaningful change could be expected in the interpretation of our results. In case of CRP, sensitivity analysis revealed that the overall pooled serum level and heterogeneity did not vary meaningfully after excluding any individual study (Fig. S12).

Five studies [32, 55, 58, 59, 62] were evaluated with the Checklist for Case-Series, and 21 studies [26–31, 33, 37, 38, 41, 51–54, 56, 57, 60, 61, 63–65] were evaluated with the Checklist for Cohort Studies. The tool evaluates articles across different domains, with each domain rated as yes, no, unclear, or not applicable. Our critical quality appraisal of the included studies is presented in the Supporting information (Tables S2–3). Overall, all studies met the requirements of the checklists, with all of them achieving more than 70% of what is considered a low risk of bias, except one study [37], which had a moderate risk of bias. The most problematic domain in almost all cases was the missing information on the confounder factors.

## Discussion

In the present study, for the first time to our knowledge, we showed with the meta-analysis of human studies that fever on hospital admission is associated with a higher death rate in AP. A significant positive correlation between admission T_b_ and mortality was also revealed with meta-regression. The presence of fever on admission in AP was accompanied by longer hospitalization, which also showed strong positive correlation with T_b_, as well as by higher clinical severity scores. Serum amylase and CRP levels were not different between the febrile and afebrile AP patient groups.

Systemic inflammation is usually accompanied by changes in T_b_ (fever or hypothermia), which is associated with the outcome of the disease in sepsis [19]. In infection-induced systemic inflammation, the change in deep T_b_ reflects the activities of the pro- and anti-inflammatory processes and their balance; therefore, T_b_ can indicate the severity, progression, and outcome of the disease. In sepsis, fever is associated with a reduced death ratio [25], which results foremost from the beneficial effects of a higher T_b_ on the host immune system, thereby enhancing the elimination of the pathogen. AP is also accompanied by systemic inflammation, but the pathogenesis of the inflammatory response differs from sepsis. Characteristically in AP, the immune system becomes activated by DAMPs that initiate and maintain the immune response differently from PAMPs, although common mechanisms also exist [17, 69, 70]. In addition to the inflammatory response, DAMPs can also mediate tissue repair [71]. The balance between the inflammatory and restitution process determines the progression of the disease and the outcome.

An increased T_b_ in AP may potentially result from an infectious comorbidity or an early superinfection, both of which could worsen the outcome of the disease, but unfortunately, we could not analyze their potential effect in the present study due to data unavailability. However, primary infections are among the rare causes of AP [72, 73], while infectious complications (e.g., abdominal sepsis, bacteremia) in AP occurred only at medians of 4–8 days after admission in different studies [74, 75]. Pancreatic infection, which is usually linked to the development of pancreatic necrosis, develops in even later stages of the disease [67, 76]. Therefore, it is not likely that infection-related T_b_ changes were present in the majority of the studies and meaningfully influenced our results. The contribution of distinct underlying mechanisms to the thermoregulatory changes in AP and sepsis is also supported by our finding that in AP the presence of fever on admission was associated by increased mortality, whereas in sepsis fever is typically accompanied with reduced death rate [77].

It should be also mentioned that bradykinin levels are increased in AP as a result of acinar cell necrosis, which may contribute to the release of proinflammatory (including pyrogenic) cytokines such as interleukins and tumor necrosis factor-α (as reviewed by Petersen and colleagues [78]). Bradykinin activation of stellate cells causes further damage of the acinar cells, thereby creating a necrotic amplification loop in AP [78]. Interestingly, there is evidence indicating that bradykinin increases uncoupling protein 1 expression and thermogenesis in brown adipose tissue [79], which may serve as a plausible mechanism of the higher T_b_ in AP.

An alternative explanation for our findings can be that the Toll-like receptor (TLR) and nucleotide-binding oligomerization domain-like receptor (NLR) families, which are the major contributors to inflammation in AP, can be both activated by DAMPs [80]. The NLR pyrin domain-containing protein 3 (NLRP3) inflammasome was shown to mediate the parallel activation of the systemic inflammatory response and the compensatory anti-inflammatory response syndrome in experimental models of severe AP [17]. A recent meta-analysis revealed that inhibition of the NLRP3 inflammasome has protective effects in AP by attenuating organ injury and systemic inflammation in animal studies, indicating that NLRP3 inflammasome offers good prospects as a target for specific AP therapy [81]. Importantly, NLRP3 is also linked to thermoregulation, since mutations in the genes of NLRP3 lead to hereditary periodic fever syndromes [as reviewed by Zhong and colleagues [82]], which indicates its role in febrigenesis.

The high mobility group box 1 (HMGB1) protein can be released by injured or dying cells, hence serving as a DAMP to initiate non-infectious inflammation [69]. Similarly to bacterial endotoxin, HMGB1 binds to TLR4, but it activates different gene expression patterns compared to endotoxin [83, 84]. HMGB1 is also involved in the development of fever, anorexia, and sickness syndrome in vivo [85, 86], and plays a role as a late mediator of lethality. It was shown that serum HMGB1 levels were significantly elevated in patients with AP on admission and were correlated with the severity and mortality of the disease [87, 88]. Similar results were observed in preclinical animal models of AP [89]. In experimental models of AP, the blockade of HMGB1 reduced the severity of the disease and ameliorated associated organ damage [90–92]. The complex role of HMGB1 in AP is supported by the findings that extracellular HMGB1 released from innate immune cells aggravated the inflammatory response and increased pancreatic damage, whereas pancreatic-derived intracellular HMGB1 limited the severity of the disease in mice [93].

The diverse contributions of the NLRP3 inflammasome, HMGB1, and their potential interaction to the pathomechanism of AP have been demonstrated in multiple studies [as reviewed by Ferrero-Andres and colleagues [80]]. Unfortunately, we could not find any studies on AP in which T_b_ of the rats or subjects was measured. Nevertheless, it is well documented that NLRP3 and HMGB1 are both associated with various febrile diseases in humans [94–103], wherein a biomarker role for disease severity and prognosis was also suggested for HMGB1 [104–106]. Moreover, the blockade of HMGB1 with antibodies reduced T_b_ in experimental models of systemic inflammation [107].

Based on the above-described mechanisms, it seems reasonable to assume that the association of fever on hospital admission with the increased severity and mortality of AP can be explained by an excessive bradykinin and DAMP-induced activation of the inflammatory response in AP, which overwhelms the compensatory anti-inflammatory response, thereby leading to further tissue damage of the host and then to the release of more bradykinin and DAMPs, thus creating the vicious cycle. The development of the vicious cycle is indicated by the higher T_b_ (as a part of the overt activation of the inflammatory response) and worsens the severity of the disease, eventually leading to fatal outcome. We did not find an association between T_b_ and serum amylase or CRP levels; however, the accuracy of their absolute levels for predicting disease severity is questionable at the onset of AP [108–111].

Our meta-analysis included data from a total of 40,630 AP patients with overall 906 mortality events. We believe that our search strategy was broad enough and included the three main databases of human studies; consequently, 26 full-text articles could be involved in our analyses. The overall sample size and death rate can be considered large enough to draw conclusions about the association between T_b_ and mortality rate in AP, but limitations of our study should also be mentioned.

We could not assess the potential importance of AP-induced hypothermia, because we did not find any reports including patient groups with a T_b_ of less than 36°C on hospital admission. This might be due to the low number of hypothermic individuals in the patient groups, but it is also possible that—unlike in sepsis, in which a decreased T_b_ develops in 9–16% of the patients [19]—spontaneous hypothermia is not characteristic even in severe forms of AP. As a methodological concern regarding clinical thermometry, it should be noted that the sites and devices of T_b_ measurement were not reported in any of the analyzed studies. This could lead to undiagnosed cases of fever in the AP patients, while it could also increase heterogeneity because big differences between different methods (e.g., rectal vs axillary) were reported [42, 112]. We could not find any papers reporting regularly repeated T_b_ measurements, hence we could not assess the T_b_ dynamics of the patients. Furthermore, analyzable data on the use of antipyretic drugs before the T_b_ measurement could not be extracted from the studies, which could have clearly affected our results. It is, however, highly unlikely that the absence of fever was due to antipyretics in all 16 study groups of the 39,912 patients, and that antipyretic drugs were not used in any of the 12 study groups of the 718 febrile patients. It would have been also important to perform subgroup analysis based on the different etiologies of AP; however, eligible data on T_b_ and the outcomes of interest in the patient groups with different etiologies were not reported in the studies.

We were able to show significant associations between deep T_b_ and outcome measures in AP; however, the underlying mechanisms that could connect fever with worse outcome could not be investigated in our study. We could only discuss several potential mechanisms, which may or may not be involved in the explanation of the showed associations. Hence, the discovery of the exact mechanism for the explanation of our findings remains subject for future research.

With the exception of 4 studies [41, 56, 60, 64], the majority of the studies (*n* = 22) included in our meta-analysis were single-center studies, with designs ranging from retrospective [27–29, 31–33, 54, 55, 57, 59, 61, 65] to prospective [30, 37, 51, 52, 58, 62, 63] to randomized clinical trials [26, 38, 53]. According to our quality assessment, 25 studies were considered as good quality with low risk of bias [26–33, 38, 41, 51–65], only one study considered to have a moderate risk of bias [37] according to the JBI Critical Appraisal Checklist for Case-Series and Cohort Studies. In part because of the aforementioned reasons, the studied patient populations were quite diverse; methodological and medical differences in study design could all contribute to the considerably high between-study heterogeneity observed in our analysis. To account for the presence of heterogeneity, we used the random-effects model in all forest plots of our meta-analyses.

## Conclusion

Despite the mentioned limitations, our meta-analysis suggests that T_b_ on hospital admission has prognostic value for predicting the severity and the outcome of AP. Our findings warrant the need for accurate and regular T_b_ measurements already from the beginning of the disease. Whether the increased T_b_ is an indicator of disease severity (via bradykinin/DAMP-related mechanisms as suggested above) or a causative factor of the enhanced progression of AP could not be answered in the present work due to its meta-analysis nature; thus, it remains subject for future studies. Our results can also serve as a basis for the design of high-quality clinical studies with standardized T_b_ measurements aiming to determine the prognostic performance of admission T_b_ and T_b_ dynamics in patients with AP.

## Supplementary Information

Supplementary Material 1: Table S1: PRISMA checklist. Fig S1: Funnel plot to Fig. 2. Fig S2: Effect size and heterogeneity sensitivity analysis to Fig. 2. Fig S3: Funnel plot to Fig. 4. Fig S4: Effect size and heterogeneity sensitivity analysis to Fig. 4. Fig S5: Funnel plot to Fig. 6. Fig S6: Funnel plot to Fig. 7. Fig S7: Effect size and heterogeneity sensitivity analysis to Fig. 6. Fig S8: Effect size and heterogeneity sensitivity analysis to Fig. 7. Fig S9: Funnel plot to Fig. 8. Fig S10: Funnel plot to Fig. 9. Fig S11: Effect size and heterogeneity sensitivity analysis to Fig. 8. Fig S12: Effect size and heterogeneity sensitivity analysis to Fig. 9. Table S2: JBI Checklist for Case-Series. Table S3: JBI Checklist for Cohort Studies.

## Data Availability

All data generated or analyzed during this study are included in this published article and its supplementary information files.

## Abbreviations

AP: Acute pancreatitis
APACHE II: Acute Physiology and Chronic Health Evaluation
CI: Confidence interval
CRP: C-reactive protein
DAMP: Damage-associated molecular pattern
HMGB1: High mobility group box 1
LOS: Length of hospital stay
NLR: Nucleotide-binding oligomerization domain-like receptor
NLRP3: Nucleotide-binding oligomerization domain-like receptor pyrin domain-containing protein 3
PAMP: Pathogen-associated molecular pattern
PRISMA: Preferred Reporting Items for Systematic Reviews and Meta-Analyses
SD: Standard deviation
SE: Standard error
SOFA: Sequential Organ Failure Assessment
T_b_: Body temperature
TLR: Toll-like receptor
